# Performance Exploration of Ni-Doped MoS_2_ in CO_2_ Hydrogenation to Methanol

**DOI:** 10.3390/molecules28155796

**Published:** 2023-08-01

**Authors:** Yongning Yuan, Liyue Qi, Zhuxian Gao, Tuo Guo, Dongdong Zhai, Yurong He, Jingjing Ma, Qingjie Guo

**Affiliations:** 1 State Key Laboratory of High-Efficiency Coal Utilization and Green Chemical Engineering, College of Chemistry and Chemical Engineering, Ningxia University, Yinchuan 750021, China; 2 Guangdong Bangpu Recycling Technology Co., Ltd., Foshan 528000, China; 3 College of Chemical Engineering, Qingdao University of Science & Technology, Qingdao 266042, China

**Keywords:** CO_2_ hydrogenation, S vacancies, methanol selectivity, MoS_2_/Ni_0.2_

## Abstract

The preparation of methanol chemicals through CO_2_ and H_2_ gas is a positive measure to achieve carbon neutrality. However, developing catalysts with high selectivity remains a challenge due to the irreversible side reaction of reverse water gas shift (RWGS), and the low-temperature characteristics of CO_2_ hydrogenation to methanol. In-plane sulfur vacancies of MoS_2_ can be the catalytic active sites for CH_3_OH formation, but the edge vacancies are more inclined to the occurrence of methane. Therefore, MoS_2_ and a series of MoS_2_/Ni_x_ and MoS_2_/Co_x_ catalysts doped with different amounts are prepared by a hydrothermal method. A variety of microscopic characterizations indicate that Ni and Co doping can form NiS_2_ and CoS_2_, the existence of these substances can prevent CO_2_ and H_2_ from contacting the edge S vacancies of MoS_2_, and the selectivity of the main product is improved. DFT calculation illustrates that the larger range of orbital hybridization between Ni and MoS_2_ leads to CO_2_ activation and the active hydrogen is more prone to surface migration. Under optimized preparation conditions, MoS_2_/Ni_0.2_ exhibits relatively good methanol selectivity. Therefore, this strategy of improving methanol selectivity through metal doping has reference significance for the subsequent research and development of such catalysts.

## 1. Introduction

In the process of rapid human development, the excessive emission of carbon dioxide has led to a series of natural disasters such as global warming, continuous rise in sea levels, agricultural production reduction, and growing ocean acidification [1,2,3]. It is noteworthy that CO_2_ can be described as a cheap and abundant C1 material if carbon dioxide is captured by a reasonable means and formed into chemical products with high industrial application value (methanol, aromatics, etc.) [4,5,6]. Among the abundant chemicals mentioned above, methanol is the preferred choice for the direct catalytic reaction of CO_2_ gas to oxygenates because it can be considered an excellent precursor for some chemicals and a fuel substitute [7,8]. The efficient catalysis of CO_2_ to CH_3_OH is exothermic, and low temperatures favor equilibrium shifts in the product direction. However, the activation of the C–O inert bond in CO_2_ requires a high temperature [9]. Therefore, considering the thermodynamic and kinetic contradictions of this reaction, it is extremely important and urgent to prepare a reasonable catalyst for the conversion of CO_2_ and hydrogen into methanol under low-temperature conditions.

The catalyst systems mainly include Cu-based metal catalysts [10,11,12], precious metal catalysts [13,14], and metal oxide catalysts [15,16,17]. As is well known, with the birth of the industrialized Cu/ZnO/Al_2_O_3_ catalyst, copper-based catalysts are the most mature and deeply explored in the research field of CO_2_ to methanol. However, the active phase is poisoned and inactivated due to the H_2_O generated by the competitive side reaction of RWGS as well as the sulfur contained in the feed gas, which ultimately leads to poor stability. Precious metal catalysts are not suitable for large-scale applications due to their high price. Therefore, it is urgent to explore relevant catalysts with high conversion rates and high stability suitable for CO_2_ to methanol under a low-temperature reaction environment.

As a two-dimensional layered material similar to graphene, the surface atoms of MoS_2_ are almost completely exposed, so the atom utilization rate is greatly improved. The modifiability is strong, and the physical compatibility is high. There is a strong covalent bond between sulfur and molybdenum atoms, and the structure is stable. The H_2_ adsorption on the MoS_2_ surface is irreversible at low temperatures, indicating that MoS_2_ has a certain hydrogen storage capacity. Because of its structural advantages, MoS_2_ shows good chemical properties, so it has been widely applied in various catalytic areas such as electrochemistry, photochemistry, and thermochemistry. Combined with the structural similarity and strong conductivity of graphene, MoS_2_ supported on graphene shows better catalytic properties for CO_2_ to methane, and the selectivity of the main product is 95% [18,19]. The catalytic mechanism and performance of the Mo_6_S_8_ cluster for the chemical reaction between CO_2_ and H_2_ to obtain CH_3_OH are explored by density functional theory (DFT). Calculation results prove that the C–O cleavage contained in H_x_CO intermediates can be effectively promoted by MoS_2_ [20]. Thus, catalyzed by MoS_2_, the high selectivity of the methanol is explained. It is found that the CO_2_ conversion is 12.5% at a low temperature of 180 °C catalyzed by the novel MoS_2_. The selectivity to methanol is high (94.3%), and the stability is maintained for 3000 h without inactivation [21]. The study shows that the sulfur vacancies contained in the basal plane are conducive to methanol formation, while the edge sulfur vacancies are the active centers for the excessive hydrogenation of methane. From this perspective, edge S vacancies are meaningful for describing the CH_3_OH selectivity, and minimizing the generation of sulfur vacancies at edge locations will be a promising strategy for improving the methanol selectivity catalyzed by MoS_2_.

Metal doping is an effective and feasible method for improving the performance of catalysts for various catalytic reactions. In addition to being directly used as a reasonable catalyst for producing methanol from CO_2_ gas, MoS_2_ can also be used as a single-atom support. The Pt monomer supported by MoS_2_ that facilitates the methanol selectivity is 95.4% because of the synergistic impact between molybdenum disulfide and adjacent Pt monomers [22]. DFT analysis shows that the Co adatom-induced interstitial states play a major role in the breakage of CO_2_ into the product methanol. Therefore, it is theoretically inferred that a single Co atom supported on a monolayer of molybdenum disulfide is an excellent catalyst for catalyzing carbon dioxide into methanol. This work proves, from the perspective of theoretical knowledge, that single-atom catalysts are supported on MoS_2_ [23]. Encouraged by these reported results, herein, a kind of nanoscale MoS_2_/Ni and MoS_2_/Co catalysts with different proportions are prepared by hydrothermal method. Experimental exploration shows that the atomic doping of Ni and Co causes the structures of MoS_2_ monolayers to be confined with NiS_2_ and CoS_2_ that are formed by Ni, Co, and S, respectively. The embedded NiS_2_ and CoS_2_ can prevent the CO_2_ and H_2_ from approaching the edge sulfur vacancy, so the CH_3_OH selectivity is improved. As an experimental result, the optimal catalysis MoS_2_/Ni_0.2_ achieved a good methanol selectivity of 83.7%. Therefore, the methanol selectivity can be improved by the metal doping strategy.

## 2. Results and Discussions

### 2.1. Exploration of Various Characterization Results

**Figure 1:**
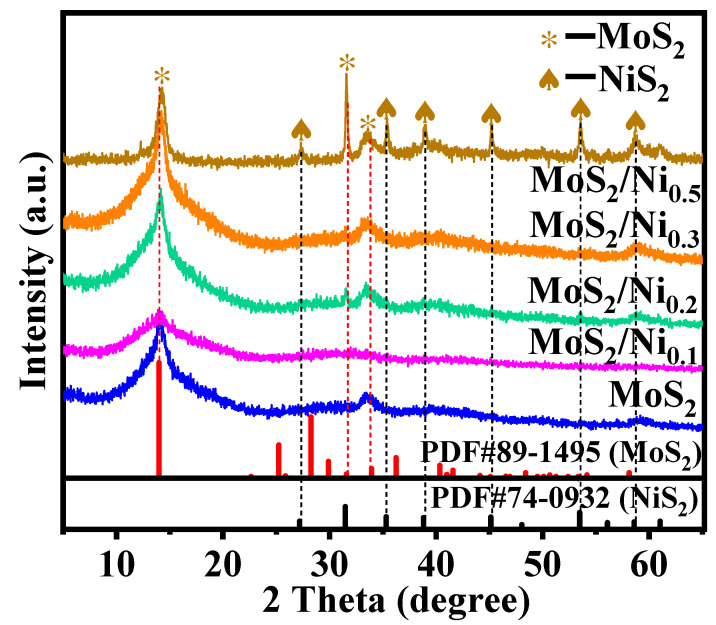
XRD results of MoS_2_/Ni_x_ (x is the addition amount of Ni(NO_3_)_2_·6H_2_O; x = 0, 0.1, 0.2, 0.3, 0.5 mmol). Blue, pink, green, orange, brown indicate MoS_2_; MoS_2_/Ni_0.1_, MoS_2_/Ni_0.2_, MoS_2_/Ni_0.3_, MoS_2_/Ni_0.5_.

As depicted in Figure 1, the crystalline phase structure of pure MoS_2_ and Ni-doped samples is confirmed by XRD patterns. The pure MoS_2_ catalyst exhibits a stable hexagonal 2H-type crystal structure when the metal Ni is undoped, and the individual diffraction peaks corresponding to the (002) (100) (103) (110) crystal planes of PDF#89-1495 are visible. All illustrations are presented in the form of broad reflections of lower intensity, indicating the poor crystallinity of the coherent scattering in the nanometer range. Ni doping makes the (002) crystal plane of MoS_2_ broaden obviously, and the diffraction peak related to Ni is not observed, indicating that the excellent dispersibility of Ni doping is conducive to the reduction of catalyst grain size. The reduction of layers number in the MoS_2_ stack after metal doping can be explained by this important result [24]. In other words, the growth of MoS_2_ grains can be effectively suppressed by metal doping. As the doping amount of Ni increases to 0.2 mmol, the diffraction peak of MoS_2_ is enhanced, and a peak of NiS_2_ appears, which is because of the rapid binding of Ni ions with S^2−^ that is produced by the decomposition of thiourea. With the further increase in Ni doping amount, the sharp diffraction peaks and smaller half widths of NiS_2_ and MoS_2_ indicate that their crystallinities become stronger and the grain sizes are increased. Different from Ni doping, because the electronegativity of the Co element is weaker than Ni in the same period, the chemical forces between Co and MoS_2_ are relatively weak, resulting in a worse dispersibility of Co doping than Ni, so the (002) crystal plane of MoS_2_ is higher than Ni doping (Figure S1).

**Figure 2:**
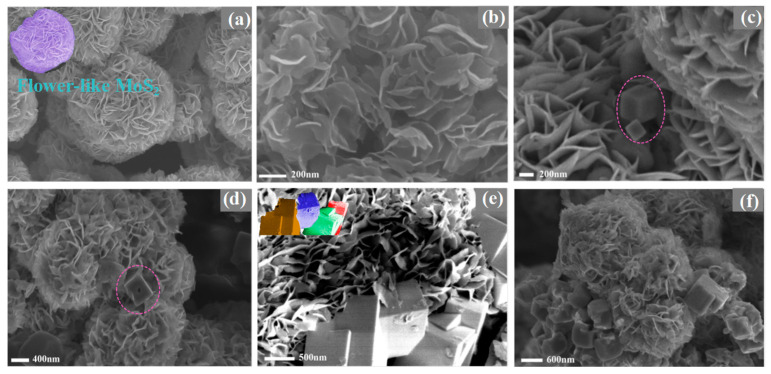
SEM photograph of MoS_2_/Ni_x_ (x is the addition amount of Ni(NO_3_)_2_·6H_2_O; x = 0, 0.1, 0.2, 0.3, 0.5 mmol). (**a**) MoS_2_/Ni_0_, (**b**) MoS_2_/Ni_0_, (**c**) MoS_2_/Ni_0.1_, (**d**)MoS_2_/Ni_0.2_, (**e**) MoS_2_/Ni_0.3_, (**f**) MoS_2_/Ni_0.5_. The circles indicate NiS_2_.

To further demonstrate the apparent structures of pure MoS_2_ and the new catalysts formed by metal doping, scanning electronic microscopy (SEM) is analyzed. In MoS_2_ structure, six S atoms are tightly packed up and down to form a sixfold triangular prism, and the triangular prism voids are formed. Mo atoms are filled into these voids, resulting in a two-dimensional sheet. Monolayers of 2D flakes can stack through van der Waals forces and weak coordination bonds and form different types of interlayer voids. Therefore, as shown in Figure 2a,b, MoS_2_ is composed of abundant lamellar nanoflowers, and there are certain gaps between the layers, which is beneficial for deep adsorption of carbon dioxide gas and hydrogen with the catalyst. As depicted in Figure 2c, compared to pure MoS_2_, a small amount of doped Ni ions is quickly combined with the S^2−^ that is generated by the decomposition of thiourea to form larger NiS_2_ particle cubes, with a trend of concave surfaces along the centerline. As the reaction progresses, the raw materials continue to react with these large cubes, forming newly exposed surfaces from the centerline of the cube surface and gradually extending towards the interior of the cube, so the smaller NiS_2_ volume cube particles are formed and separated. 

When the Ni doping amount increases to 0.2 mmol, only irregular small particles are observed and are just filled at the outer edge of the MoS_2_ layer, resulting in an increasing in interlayer spacing and a reduction of layers number, so the particles are reduced (Figure 2d), which are consistent with the XRD results. On the one hand, the stacking height and voids are increased, and the multilayer stacking structure is more stable [25]. On the other hand, the benign increase in interlayer spacing is conducive to the deep adsorption of carbon dioxide and hydrogen molecules between the layers, the location of NiS_2_ leads to the exposure of less edge S vacancies, and, finally, the CH_3_OH selectivity is effectively promoted. With further doping of Ni, the sample exhibits unseparated cubic blocks or irregular particles, a large amount of NiS_2_ is accumulated and covers the surface of MoS_2_, and the CH_3_OH selectivity is reduced (Figure 2e,f). Therefore, the formation of NiS_2_ particles is an anti-Ostwald ripening evolution model of large particle splitting and small particle growth separation [26]. An appropriate amount of metal doping can effectively improve the CH_3_OH selectivity. On the contrary, excessive metal addition will cause a large area of NiS_2_ accumulation in the material surface layer, which will affect the catalytic performance. Similar situations have also occurred with Co doping (Figure S2).

**Figure 3:**
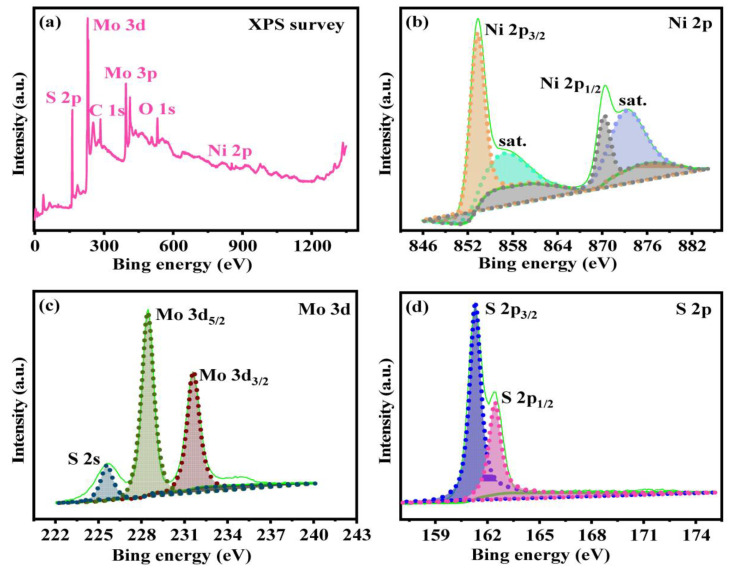
XPS spectrum of MoS_2_/Ni_0.2_ catalyst. (**a**) XPS survey, (**b**) Ni 2p, (**c**) Mo 3d, (**d**) S 2p.

The chemical states of elements of the best-performing MoS_2_/Ni_0.2_ catalyst are measured through X-ray photoelectron spectroscopy (XPS) for further study. The XPS measured spectra are shown in Figure 3, and all surface composition values are obtained from the spectral results. As displayed in Figure 3a, the Mo, S ratio in MoS_2_ conforms to the chemical formula, while the ratio of these two elements in MoS_2_/Ni_0.2_ is lower than the chemical formula. The spectrum of Ni 2p is composed of spin-orbit peaks at 853.2 eV and 870.8 eV of Ni 2p_3/2_ and Ni 2p_1/2_, respectively. An oscillatory satellite peak occurs from the Ni element, displaying the presence of Ni^2+^ (Figure 3b) [27]. The XPS spectrum related to Mo 3d region is displayed in Figure 3c. The binding energies at 232.0 eV and 229.1 eV are attributed to Mo 3d_3/2_ and Mo 3d_5/2_, respectively, proving that Mo appears with Mo^4+^ valence state in MoS_2_/Ni_0.2_. The characteristic peak at 226.1 eV corresponds to S 2s. This phenomenon is in agreement with the spectral phenomena of the Mo 3d region of molybdenum disulfide without Ni doping, which proves that the doping of Ni does not result in a fundamental change in the valence state of molybdenum and the successful formation of MoS_2_ in MoS_2_/Ni_0.2_ [28]. It is worth mentioning that the appearance of the Ni–Mo chemical bond confirms that there is a strong chemical force between MoS_2_ surfaces and Ni in the MoS_2_/Ni_0.2_ catalyst, which may weaken the Mo–S bond. XPS shows two strong S 2p peaks corresponding to binding energies at 163.2 eV as well as 161.8 eV for the S 2p_1/2_ and S 2p_3/2_ binding energies of S^2−^ in the MoS_2_/Ni_0.2_ sample [29,30,31], which is attributed to the low surface coordination of sulfide ions and the lattice S of metal–sulfur bonds in NiS_2_ (Figure 3d) [32]. Therefore, the doping of Ni leads to the charge-density redistribution, the density of edge S vacancies is decreased, and the CH_3_OH selectivity is improved [33].

**Figure 4:**
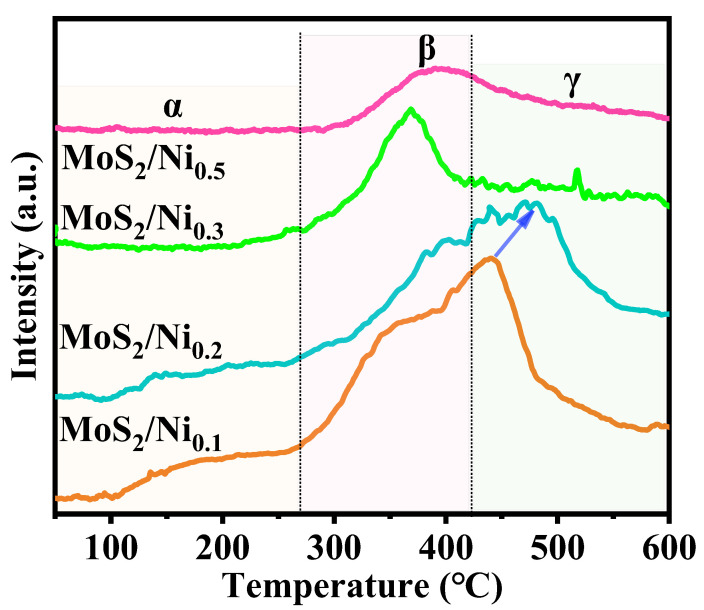
CO_2_ adsorption characteristic curve of MoS_2_/Ni_x_ (x is the addition amount of Ni(NO_3_)_2_·6H_2_O; x = 0.1, 0.2, 0.3, 0.5 mmol).

The chemical adsorption and effective catalysis of carbon dioxide are related to the alkalinity of the MoS_2_ surface [34,35]. To further explore the role of doped metals in the CO_2_ adsorption characteristics, the CO_2_–TPD results of MoS_2_/Ni_x_ (x = 0.1, 0.2, 0.3, 0.5) are shown in Figure 4. By comparing the desorption peak areas and peak positions of the four different Ni-doped catalysts, these materials have a certain adsorption capacity for acidic CO_2_ molecules. The CO_2_–TPD curve of MoS_2_/Ni_x_ (x = 0.3, 0.5) catalysts mainly contain medium-strength alkaline sites. The MoS_2_/Ni_x_ (x = 0.1, 0.2) catalyst exhibits three CO_2_ desorption regions, and the MoS_2_/Ni_0.2_ has the strongest alkaline site. The strong alkaline sites are closely related to methanol selectivity, which may lead to the MoS_2_/Ni_0.2_ catalyst having the best methanol selectivity [36]. As the amount of Ni added further increases, the desorption peak slowly becomes flat and wider, indicating that the bonding ability between CO_2_ and the catalyst is gradually weakened, and the catalytic performance decreased accordingly. This result indicates that an appropriate amount of Ni is doped on MoS_2_, with the effective acid–base interaction between the basic S–Mo and S–Ni functional groups of the catalyst and the acidic CO_2_ molecules. In addition, the dipole–dipole chemical interaction between carbon dioxide and the polar sites related to S functional groups also plays a key role in the adsorption level of CO_2_ gas, which has a positive effect on improving product selectivity [37]. 

However, excessive Ni doping results in ineffective contact between CO_2_ and catalyst sites. Interestingly, the peak shapes and peak areas of materials are also different with different Ni doping amounts, indicating that the CO_2_ adsorption is regulated by both the active component content and the MoS_2_ support. Similarly, the CO_2_–TPD curves of MoS_2_/Co catalysts prepared under the same conditions with different Co doping levels are shown in Figure S3. From the comparison chart of the adsorption level of the CO_2_ molecule, it can be seen that the four samples all have the corresponding desorption peaks in the intermediate and high-temperature regions. Although the strong alkaline site of MoS_2_/Co_0.2_ moves towards the high-temperature zone, the medium alkaline site moves towards the low-temperature zone. Thus, through CO_2_–TPD analysis, it can be deduced that MoS_2_/Ni_0.2_ is an ideal catalyst for carbon dioxide hydrogenation to methanol, which must be confirmed by activity evaluation.

**Figure 5:**
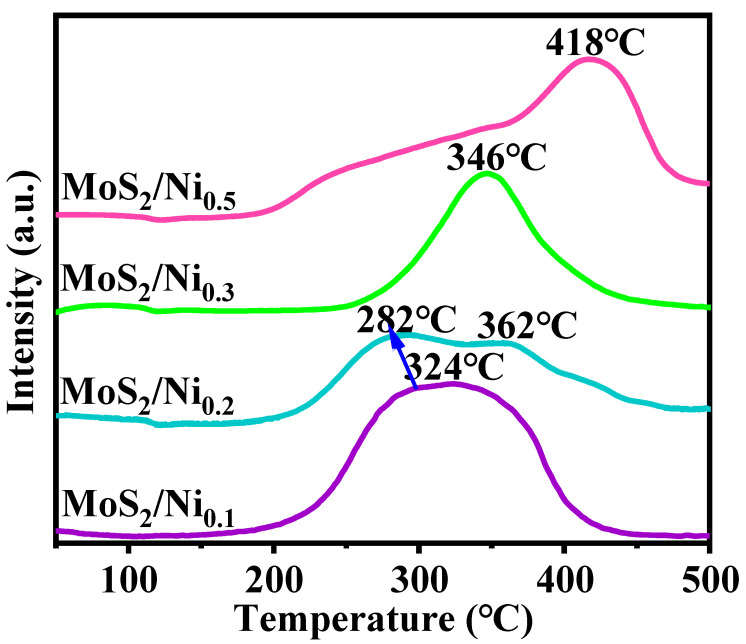
H_2_–TPR reduction curve of MoS_2_/Ni_x_ (x is the addition amount of Ni(NO_3_)_2_·6H_2_O; x = 0.1, 0.2, 0.3, 0.5 mmol).

The physical adsorption of hydrogen molecules on the catalyst surface is replaced by effective chemical adsorption at a certain temperature. Therefore, as shown in Figure 5, the reduction performance of the catalyst is studied through H_2_–TPR experiments. The active hydrogen rapidly migrates between adjacent S atoms to form H–S–H and the Mo or S edge is gradually reduced through “dissociation diffusion” [38,39]. Because of different particle shapes and dispersion levels in MoS_2_, different Ni doping differs in adsorption temperature and intensity. For MoS_2_/Ni_0.1_, one main peak appears at 324 °C [40]. With the suitable increase in metal doping, the double active sites are gradually enhanced, and the reduction energy consumption decreases, so that the reduction peaks of H_2_–TPR shift to the low-temperature direction. After the Ni doping amount is increased to 0.2 mmol, two well-separated peaks appear at 282 °C and 362 °C; the first peak significantly moves to lower reduction temperatures, and MoS_2_/Ni_0.2_ has the lowest reduction temperature, indicating that the metal sulfur bonds energy is reduced. 

Combining XRD and SEM analysis, it is found that the strong interaction between doped metals and MoS_2_ is conducive to the dispersion of the catalyst and the reduction of catalyst particle size. The increased interlayer spacing of MoS_2_ results in an increase in Ni/Mo edge site. Hydrogen participates in the surface reaction, and the sulfur atom is reductively removed, resulting in an irreversible change of MoS_2_ structure. Thus, the Mo–S bond strength is weakened, the reducibility of MoS_2_ is enhanced, and a good reaction basis for the carbon dioxide hydrogen to methanol is provided [40]. The doping amount of Ni increased to 0.3 and 0.5 mmol, and the excessive and irregular accumulation of NiS_2_ is covered on the MoS_2_ surface. The reduction temperature is increased, and the reduction ability of the catalyst is weakened. Compared with MoS_2_/Co_x_ catalysts, the MoS_2_/Co_0.2_ catalyst also has a good downward shift of the first peak position, but the reduction temperature is still higher than MoS_2_/Ni_0.2_ (Figure S4). Thus, the catalytic performance of Co-doped MoS_2_ may be lower than that of MoS_2_/Ni_X_.

**Figure 6:**
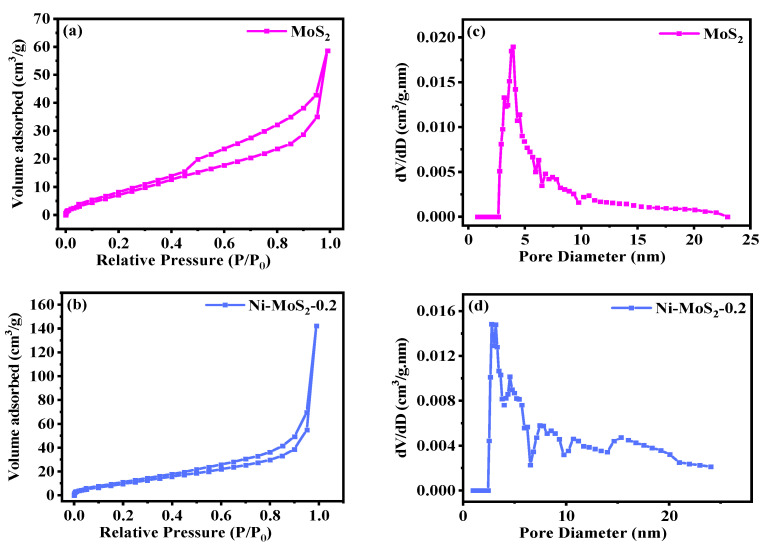
N_2_ desorption curve (**a**,**b**) and pore size distribution (**c**,**d**) of MoS_2_ and MoS_2_/Ni_0.2_.

The specific surface area and pore size distribution of the material play an important role in the performance of the catalyst. The MoS_2_/Ni catalyst with Ni doping amount of 0.2 mmol and MoS_2_ catalyst are analyzed by N_2_ physical adsorption and desorption technology. The N_2_ adsorption and desorption trend and pore size distribution characteristics are listed in Figure 6 and Table 1. The adsorption isotherms of the MoS_2_ catalyst and MoS_2_/Ni_0.2_ catalyst belong to class VI isotherms. According to the standard classification of IUPAC, the characteristics of such isotherms reflect the characteristics of mesoporous materials (Figure 6a,b). When the relative pressure of P/P_0_ of MoS_2_ is 0.45~1.0, and the relative pressure of P/P_0_ of MoS_2_/Ni_0.2_ is 0.8~1.0, the adsorption capacity is improved. The pore size distribution ranges of the involved materials are shown in Figure 6c,d, which are obtained from the analysis of the pore size distribution curve. The highest point corresponding to the pore radius is greater than 2 nm, which further proves that both MoS_2_ and MoS_2_/Ni_0.2_ catalysts are mesoporous materials. Compared with MoS_2_, the MoS_2_/Ni_0.2_ catalyst exhibits a relatively smaller specific pore diameter as well as pore volume. It can be seen that the excellent CH_3_OH selectivity of MoS_2_/Ni_0.2_ may be due to its relatively small pore volume (0.090 cm^3^·g^−1^) and pore size (10.13 nm).

### 2.2. Performance of CO_2_ Feed Gas to CH_3_OH Conversion Catalyzed by MoS_2_, MoS_2_/Ni_x_, and MoS_2_/Co_x_

**Figure 7:**
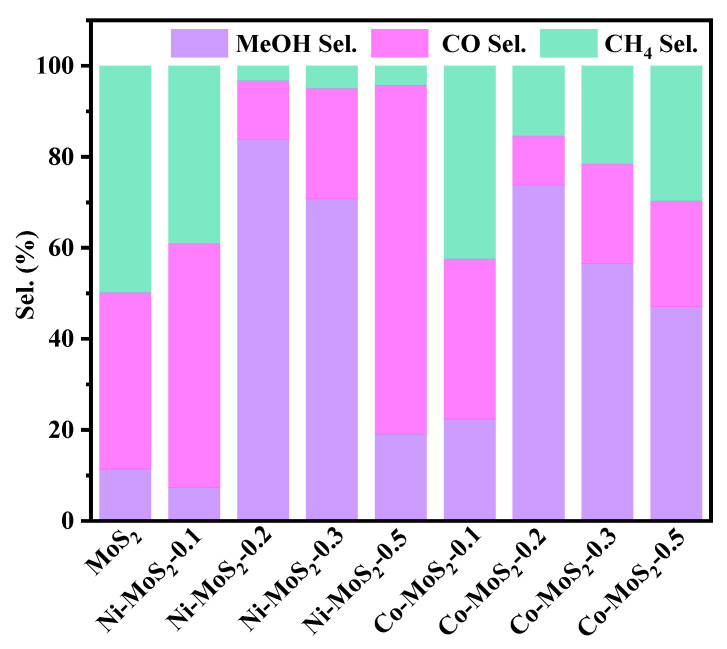
CH_3_OH selectivity of MoS_2_, MoS_2_/Ni_x_, and MoS_2_/Co_x_ catalysts.

Under the condition settings of 260 °C, 5 MPa, and 12,000 mL h^−1^ g_cat_^−1^ of gas hourly space velocity (GHSV), all catalytic evaluations are performed by a fixed-bed reactor, and the experimental results after testing are listed in Figure 7. As a comparative catalyst, the property of 2H-type MoS_2_ is studied first. The result shows that the CO_2_ conversion rate is 3.36% (Figure S5) and the methanol selectivity is 11.34%, and the main byproducts are methane and carbon monoxide. The catalytic characteristic of MoS_2_ for CO_2_ to product is well demonstrated by the experimental results, but the conversion and selectivity are expected to be improved. With the increase in Ni and Co doping amount, the CH_3_OH selectivity is also increased. When the Ni and Co doping amount is 0.2 nmol, the CH_3_OH of the catalyst MoS_2_/Ni_0.2_ and MoS_2_/Co_0.2_ reach the highest 83.73% and 73.82% (Figure 7). However, when the doping amount is further increased from 0.2 mmol to 0.3 mmol, the methanol selectivity shows a downward trend, because the appropriate proportion of Ni and Co addition leads to the blocking effect of edge S vacancies. On the contrary, excessive Ni and Co metals are doped, a large amount of NiS_2_ and CoS_2_ are stacked and covered on the reaction surface of the catalyst, the exposed sulfur vacancy active sites are blocked, and the catalytic performance is reduced. By comparing the CH_3_OH selectivity of Ni and Co-doped catalysts, it is determined that the catalytic property of the Ni-doped MoS_2_ catalyst slightly surpasses the Co-doped MoS_2_ catalyst.

### 2.3. Intermediates and Mechanisms Properties Involved in CO_2_ Hydrogenation to Methanol 

#### 2.3.1. Determination of Intermediates through In Situ Infrared Spectroscopy

**Figure 8:**
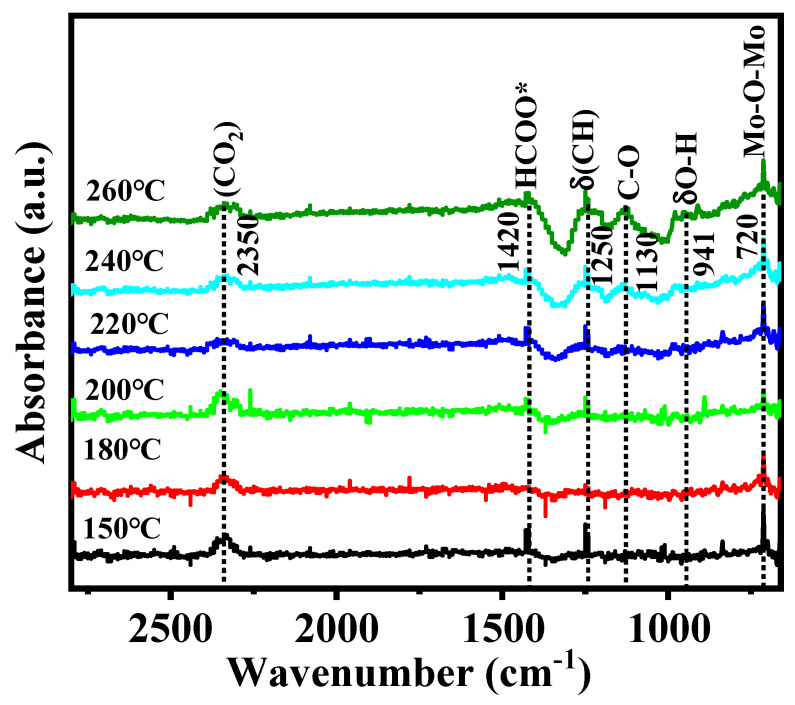
In situ DRIFTS spectra of MoS_2_/Ni_0.2_ catalyst from 150 °C to 260 °C; the feed gas is CO_2_ and H_2_ (H_2_:CO_2_ = 3:1). * represents the adsorbed species on catalyst surface.

The key adsorbents and intermediates included in the formation of methanol on the MoS_2_/Ni_0.2_ catalyst with the best performance are shown in Figure 8. The characteristic peak of CO_2_ gas appears at approximately 2350 cm^−1^ [41]. The peaks at 720 cm^−1^ and 907 cm^−1^ are attributed to O* characteristic and the peak intensity is obvious [21]. The vibration peak at 1080 cm^−1^ is attributed to the methoxy group (CH_3_O*) [42]. In situ DRIFTS experimental results show that the chemical bond of CO_2_ adsorbed on the MoS_2_/Ni_0.2_ surface is broken and forms CO* and O*, then CO* and dissociated H* react to form HCO* species. The generation of methoxy and methanol is due to subsequent gradual hydrogenation.

#### 2.3.2. Determination of Mechanisms Properties through DFT Calculation

**Figure 9:**
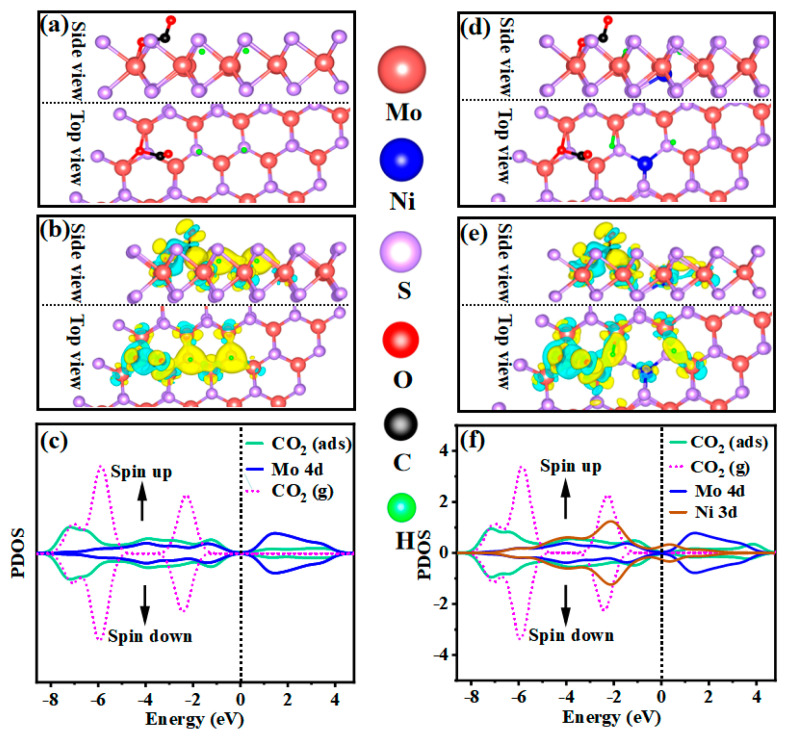
(**a**,**d**) Top and side views of the optimal adsorption structures of CO_2_ on MoS_2_ and MoS_2_/Ni. (**b**,**e**) The electron transfer between MoS_2_, MoS_2_/Ni surfaces, and CO_2_ based on differential charge density analysis. The blue and yellow colors express electron depletion and accumulation, respectively. (**c**,**f**) The PDOS of MoS_2_, MoS_2_/Ni, and CO_2_ (g represents the CO_2_ gas, ads represent the CO_2_ gas is adsorbed on MoS_2_ surface); the vertical dotted line at zero represents Fermi level.

To provide a deeper explanation of metal doping in improving methanol selectivity, the reaction mechanisms of MoS_2_ and Ni/MoS_2_ are investigated by DFT theoretical calculations. The chemical adsorption of H_2_ and CO_2_ on the catalyst surface is determined as a prerequisite for subsequent reactions. Figure 9a shows the optimal adsorption structure for CO_2_ on the catalyst. The CO_2_ angle is from 180° to 120.20° and the O–C bond is elongated from 1.25 Å to 1.38 Å. The corresponding differential charge density (Figure 9b) confirms that Mo electrons are transferred to the adsorbed CO_2_. The corresponding PDOS analysis shows that the CO_2_ molecular orbital is not only shifted to a lower energy level after being adsorbed on the catalyst surface (Figure 9c), but also overlaps with the Mo 4d state after the CO_2_ gas becomes an adsorbed state on the MoS_2_ surface, proving the CO_2_ activation. As shown in Figure 9e, Ni doping leads to more unsaturated exposure of Mo, and the formed active hydrogen is more prone to surface migration. The interaction between Ni and the charge-rich center S in NiS_2_ gives it an appropriate affinity and bonding range. The local action of negative charge on the S atom and H_2_ occurs at the highest spin density position. The activation process involves the transfer of negative charge from the S atom to the H_2_ antibonding orbital σ*, the electron cloud distribution and orbital energy of the hydrogen molecule are changed, the four-center transition state is formed, the S–S and H–H bonds are weakened and heterocleaved, and the S–H bond is strengthened, which eventually leads to the breaking of the former and the formation of the latter. A larger range of orbital hybridization near the Fermi level (Figure 9f) makes the H_2_ bond break more thoroughly (Figure 9d). Combined with in situ infrared spectroscopy, the effective dissociation of hydrogen gas is conducive to the gradual hydrogenation of CO*, so the methanol selectivity is more highly catalyzed by MoS_2_/Ni_0.2_.

**Figure 10:**
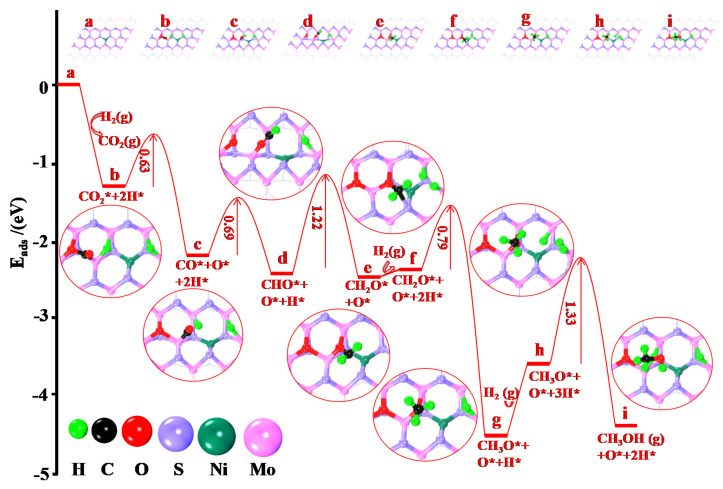
The geometry structures and adsorbed energies of CH_3_OH formation on MoS_2_/Ni surface. * represents the adsorbed species on catalyst surface.

The chemically adsorbed CO_2_ is considered to have two reduction pathways: the formate pathway and CO hydrogenation. According to in situ DRIFTS spectra, CO_2_ hydrogenation catalyzed by MoS_2_/Ni tends to an oxidation–reduction pathway, and the optimal adsorption structures of different intermediates as well as barrier energy are shown in Figure 10. CO_2_ and H_2_ are directly dissociated into CO*, O*, and 2H* through the energy barrier of 0.63 eV. The large adsorption energy indicates that the interaction between CO* and MoS_2_ is very strong (E_ads_ = −1.27 eV), and is more likely to undergo further reaction on the support without being released and forming CO gas. The calculation results indicate that CO* can be hydrogenated to CHO* by crossing only a potential barrier of 0.69 eV. It is evident that the carbon involved in CO is more susceptible to H proton attack than the oxygen atom, and forms CHO anchoring at the active site for subsequent hydrogenation reaction. The second hydrogenation step of CHO to CH_2_O needs a reaction barrier of 1.22 eV. The CH_2_O is further hydrogenated to CH_3_O, which just needs a barrier energy of 0.79 eV, and this hydrogenation process is exothermic, indicating that the CH_3_O is a beneficial intermediate. In the fourth hydrogenation reaction, CH_3_O can be reduced to CH_3_OH through an energy barrier of 1.33 eV (the decisive step of the reaction system) and easily desorbed from the catalyst surface. Therefore, based on the above mechanism exploration, methanol is the final product catalyzed by MoS_2_/Ni. Finally, the reaction mechanism is summarized as CO_2_* → CO* → CHO* → CH_2_O* → CH_3_O* → CH_3_OH.

## 3. Material and Methods

### 3.1. Materials

All the materials contained in the experiments are shown in Table 2.

### 3.2. Experimental Apparatus

All the instruments and types of equipment for experiments are shown in Table 3.

### 3.3. Preparation of MoS_2_, MoS_2_/Ni, and MoS_2_/Co Materials

MoS_2_/Ni and MoS_2_/Co are prepared by hydrothermal method. Firstly, 1 mmol ammonium molybdate tetrahydrate and thiourea (14 mmol) are transferred to distilled water (35 mL). To completely dissolve the reactants and form a homogeneous solution, the beaker containing the mixed reaction liquid should be placed on a magnetic suspension stirrer and stirred for 30 min. Different contents of nickel nitrate hexahydrate or cobalt nitrate hexahydrate are weighed and placed into the above solution to be completely dissolved. The thoroughly dissolved solution is poured into a hydrothermal kettle with Teflon-lined stainless steel, and the Teflon lid is tightly closed. The hydrothermal kettle is placed into a vacuum drying box, then reacted at 210 °C for 24 h. After the completion of catalyst preparation, the hydrothermal kettle is taken out and the temperature drops to cool down. The reacted substance is washed with deionized water 3 times and then washed again with anhydrous ethanol for the same process. Finally, the washed and centrifuged material is effectively dried by a drying oven, the drying temperature is 80 °C and the time is 12 h, and the final product sample is obtained. For experimental comparison, MoS_2_ is prepared by dissolving ammonium molybdate tetrahydrate (1 mmol) and thiourea (14 mmol) using the same preparation process as MoS_2_/Ni and MoS_2_/Co.

### 3.4. Characterization Methods

The crystal phase composition of the material is determined by a German BmkerD8 advanced X-ray diffractometer (XRD). The radiation source is Cu K_α_ rays, the tube voltage is 40 kV, and the tube current is 40 mA. The scanning range is 3–85°, and the scanning speed is 8°/min.

The specific surface area and the pore size distribution of the materials, are characterized by a Quanta Autosorb IQ automatic physical and chemical adsorption instrument. The adsorbate is N_2_, and the physical adsorption test is carried out under the condition of vacuum liquid nitrogen (−196 °C). Before the adsorption test, the samples needed to be degassed at a temperature of 200 °C for 6 h. The specific surface area is obtained by linear regression of the multipoint BET (Brunauer–Emmett–Teller) model, and the pore size distribution is obtained by the DFT model.

The microscopic morphology of the material is characterized by scanning electron microscopy (SEM) (Carl Zeiss Company, Jena, Germany) NanoPorts QUANTA250/QUANTA430.

The H_2_–TPR test is carried out on a chemisorption apparatus with automatic temperature (AutoChem II 2920) from Micromeritics Company, Georgia, USA. A 50–100 mg sample is weighed and placed in reaction tubes, and the test temperature rises from 25 °C to 300 °C with a rate of 10 °C/min for drying pretreatment, then cooled to 50 °C under He gas flow (30–50 mL/min) for 1 h. A 10% H_2_/Ar mixture is passed into the reaction tube with a fixed gas flow rate (30–50 mL/min) for 1 h to be saturated, and then switched to Ar with the same gas flow to eliminate weak physical adsorption of H_2_. Finally, the tested catalyst is heated to 500 °C in Ar atmosphere (heating rate is 10 °C/min) and desorbed, and the desorbed gas is detected by TCD.

The CO_2_–TPD test is carried out on a chemical adsorber (AutoChem II 2920) from Micromeritics, USA, which is a fully automatic temperature program. A 50–100 mg sample is weighed and placed in reaction tubes, and the test temperature is increased from 25 °C to 300 °C (heating rate 10 °C/min) for drying pretreatment, then cooled to 50 °C under He gas flow (30–50 mL/min) for 1 h. A 10% CO_2_/He mixture is passed into the reaction tube at a certain gas flow rate is 30–50 mL/min for 1 h to be saturated, and then switched to He at a certain gas flow rate of 30–50 mL/min for 1 h to eliminate weak physical adsorption of carbon dioxide on the surface. The last step involves heating the catalyst to 700 °C at a heating rate (10 °C/min) in He atmosphere and desorbed, and the desorbed gas is detected by TCD.

A Thermo Scientific K-Alpha spectrometer (Thermo Fisher Scientific, Waltham, MA, USA) used for the XPS test, and the content and valence state of catalyst surface substances are analyzed under the excitation source of Al Kα rays (hv = 1486.6 eV). A suitable amount of the pressed catalyst is connected to the corresponding sample tray in a standardized manner and placed in the sample detection chamber of the Thermo Scientific K-Alpha XPS instrument. The sample is sent to the analysis chamber when the indoor pressure of the sample chamber reaches the standard value (less than 2.0 × 10^−7^ bar). The important test conditions include spot size (400 μm), work voltage (12 kV), and filament current (6 mA).

Diffuse reflectance infrared Fourier transform spectroscopy (DRIFTS) analysis is performed by Thermo Nicolet iS20 (Thermo Fisher Scientific, Shanghai, China) which has a liquid-nitrogen-cooled mercury cadmium-telluride (MCT) detector. Firstly, the catalyst is reduced at room temperature for 30 min under a pure N_2_ (20 mL/min) atmosphere. The background spectra are obtained under N_2_ blowing. Six batches of DRIFTS characterization are performed on the pretreated catalyst. Mixed CO_2_ and H_2_ (CO_2_:H_2_ = 1:3) are introduced into the chamber at 150 °C, 180 °C, 200 °C, 220 °C, 240 °C, and 260 °C for 30 min.

### 3.5. Catalytic Performance Test

**Figure 11:**
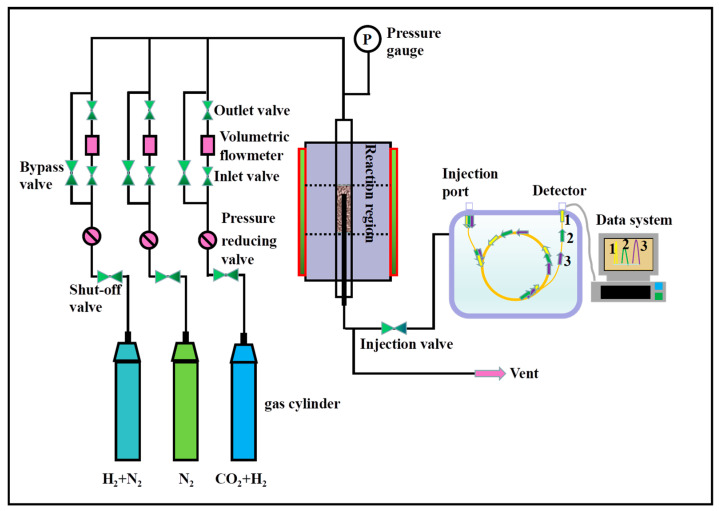
The fixed-bed reaction evaluation device.

As shown in Figure 11, the catalytic characteristic for the reaction of CO_2_ to CH_3_OH is measured on fixed-bed equipment. A certain amount of catalyst (0.2 g) and 0.4 g of quartz sand (40–60 mesh) are mixed uniformly and loaded into the constant temperature zone in the middle of the reaction tube (8 mm internal diameter). Before the performance is evaluated, the catalyst is pretreated in situ with 10 mL/min H_2_/N_2_ for 3 h under test conditions of 0.1 MPa and 300 °C. After reduction, the feed gas (H_2_:CO_2_ = 3:1) is transmitted through the reactor and fully contacts the catalyst. The subsequent reactions are performed under 5 MPa, a temperature range from 180 °C to 260 °C, and GHSV is maintained at 12,000 mL h^−1^ g_cat_^−1^. The gas phase product is detected by Agilent 8890 (Agilent Technologies (China) Co., Ltd, Beijing, China) online chromatography, and the composition and content of the product are determined according to the peak time of the product in the chromatographic column. The specific amounts of CO_2_, CO, CH_4_, and H_2_ included in the reaction product are monitored online by MolSieve 5A (Agilent Technologies (China) Co., Ltd, Beijing, China) packed column (TCD). Organic compounds such as CH_4_ and CH_3_OH in the reaction product are detected online by HP-PLOT Q-packed column (FID). 

### 3.6. Calculation Methods for Reactant Conversion as Well as Product Selectivity

The reactant conversion is calculated using the normalization method, and the formulas for CO_2_ conversion and selectivity of all products are shown in Equations (1) and (2).

(1) $$X\left({CO}_{2}\right)=\frac{f_{CO}A_{CO}+i\left(f_{{CH}_{4}}A_{{CH}_{4}}+f_{{CH}_{3}OH}A_{{CH}_{3}OH}\right)}{f_{{CO}_{2}}A_{{CO}_{2}}+f_{CO}A_{CO}+i\left(f_{{CH}_{4}}A_{{CH}_{4}}+f_{{CH}_{3}OH}A_{{CH}_{3}OH}\right)}$$

(2) $$i=\frac{f_{{CH}_{4}-TCD}A_{{CH}_{4}-TCD}}{f_{{\mathrm{CH}}_{4}-\mathrm{FID}}A_{{\mathrm{CH}}_{4}-\mathrm{FID}}}$$

(3) $$S\left({CH}_{3}OH\right)=\frac{f_{{CH}_{3}OH}A_{{CH}_{3}OH}}{f_{CO}A_{CO}+i\left(f_{{CH}_{4}}A_{{CH}_{4}}+f_{{CH}_{3}OH}A_{{CH}_{3}OH}\right)}$$

*X*(*CO*_2_): CO_2_ conversion, *Sel* (*CH*_3_*OH*): CH_3_OH selectivity, *A*: peak area, *f*: correction factor, *i*: conversion factor.

The correction factor values for each component in tail gas are shown in Table 4.

### 3.7. Calculation Methods

The Vienna Ab Initio Simulation Package (VASP) is used to calculate the relevant properties of the MoS_2_/Ni system [21]. Exchange correlation is measured through Perdew–Burke–Ernzerhof (PBE) method [43]. The plane-wave cut-off energy is 400 eV and the Monkhorst–Pack k-point sampling is 1 × 1 × 1 for the structural optimization involved in this system, while the electronic performance is calculated with the larger k-point of 3 × 3 × 1 [44]. A reasonable initial model is constructed as a 5 × 5 supercell with a vacuum thickness of 15 Å, and the size of the calculation model is 15.83 × 15.83 Å. Structural convergences are completed with Hellmann–Feynman residual force convergences between each atom below 0.02 eV/Å. A CI-NEB method is used to search the transition state involved in each elementary reaction [45]. The adsorption energy of the reactant on the catalyst surface (E_ads_) is represented by Equation (4):

E_ads_ = E_AB_ − E_A_ − E_SM_ (4)

where E_AB_ is the energy value of the reaction system where small reactant molecules are adsorbed on the material surface, E_A_ is the energy of material structure before adsorption, and E_SM_ is the energy possessed by reactant molecules adsorbed on the catalyst surface.

## 4. Conclusions

In conclusion, the MoS_2_ catalyst is prepared by hydrothermal synthesis and doped with different amounts of Ni and Co metal to obtain MoS_2_/Ni and MoS_2_/Co composite catalyst materials. The catalytic properties of these materials in CO_2_ hydrogenation to CH_3_OH are investigated.

Compared with 2H-MoS_2_, under the optimal reaction conditions, the MoS_2_/Ni_0.2_ composite catalyst shows excellent product selectivity for methanol. Thus, the high methanol selectivity from CO_2_ and H_2_ gas is achieved.

Through the analysis of XRD, XPS, BET, H_2_–TPR SEM, and CO_2_–TPD characterization results of MoS_2_, Ni/MoS_2_, and Co/MoS_2_ composite catalysts with different metal loading ratios, it is confirmed that the addition of Ni, Co metals are beneficial for blocking more edge S vacancies, and, thus, the CH_3_OH selectivity is higher. Through metal doping, the H_2_ consumption and CO_2_ adsorption are improved, indicating that the ability to activate H_2_ and CO_2_ is improved.

In situ DRIFTS spectra and DFT calculation confirmed that the hydrogenation of CO_2_ to methanol on MoS_2_/Ni catalyst follows an oxidation–reduction pathway. CO_2_ is activated into CO* and gradually hydrogenated to form methoxy groups; ultimately, methanol is generated. The improvement of CH_3_OH selectivity by metal doping involved in this paper has insight significance for subsequent related research.

## Figures and Tables

**Table 1:** Measurement results of pore structure parameters and specific surface area.

Sample	BET Specific Surface Area (m^2^·g^−1^)	Surface Pore Diameter (nm)	Pore Volume (cm^3^·g^−1^)
MoS_2_	41.70	20.65	0.214
MoS_2_/Ni_0.2_	40.73	10.13	0.090

**Table 2:** Experimental reagents and gas raw materials.

Chemical Reagent Name	Chemical Formula	Specification	Manufacturer
Cobalt nitrate hexahydrate	Co(NO_3_)_2_·6H_2_O	Analytical pure AR	Sinopharm group chemical reagent Co., Ltd. (Shanghai, China)
Nickel nitrate hexahydrate	Ni(NO_3_)_2_·6H_2_O	Analytical pure AR	Sinopharm group chemical reagent Co., Ltd.
Ammonium molybdate tetrahydrate	(NH_4_)_6_Mo_7_O_24_·4H_2_O	Analytical pure AR	Sinopharm group chemical reagent Co., Ltd.
Thiourea	CH_4_N_2_S	Analytical pure AR	Tianjin damao chemical reagent factory (Tianjin, China)
Quartz sand	SiO_2_	Analytical pure AR	Shanghai mclean biochemical technology Co., Ltd. (Shanghai, China)
Raw gas	H_2_/CO_2_/N_2_	72/24/4 (molar ratio)	Ningxia guangli comprehensive trading Co., Ltd. (Yinchuan, China)
Reducing gas	H_2_/N_2_	H_2_/N_2_ = 30/70	Beijing yanan weiye gas Co., Ltd. (Beijing, China)
High purity nitrogen	N_2_	99.999%	Ningxia guangli comprehensive Trading Co., Ltd.
High purity helium	He	99.999%	Ningxia guangli comprehensive trading Co., Ltd.
Air	Air	100%	air generator (Yinchuan, China)

**Table 3:** Instruments and types of equipment for experiments.

Equipment Name	Instrument Model	Manufacturer
Carbon dioxide catalytic conversion evaluation device	Φ240 × Φ22 × Φ510	Beijing Kunlun yongtai Technology Co., Ltd. (Beijing, China)
Gas chromatograph	Agilent GC8890	Agilent Technologies (China) Co., Ltd. (Beijing, China)
Electric heating constant temperature blast drying oven	GZX-9240MBE	Shanghai boxun industrial Co., Ltd. medical equipment factory (Shanghai, China)
Tube furnace	TL1200	Nanjing boyuntong instrument technology Co., Ltd. (Nanjing, China)
Desktop high-speed centrifuge	H/T16MM	Hunan hercy instrument equipment Co., Ltd. (Changsha, China)
Magnetic stirrer	DF-101S	Shandong juancheng hualu electric heating instrument Co., Ltd. (Heze, China)
Powder tablet press	35 mm	Hefei kejing material technology Co., Ltd. (Hefei, China)
Electronic balance	PL602	Mettler toledo Instruments Ltd. (Zurich, Switzerland)
Hydrogen generator	SPH-300	Beijing China HP analytical technology research institute (Beijing, China)
Air generator	SPB-3	Beijing China HP analytical technology research institute
Column (TCD)	HP-PLOT Q	Agilent Technologies (China) Co., Ltd. (Beijing, China)
Column (FID)	MolSieve 5A	Agilent Technologies (China) Co., Ltd.

**Table 4:** Correction factor value of components in tail gas.

Component *i*	CO_2_	CO	CH_3_OH	CH_4_-TCD	CH_4_-FID
Correction factor *f_i_*	1.06	1.24	2.13	1.66	1

## Data Availability

Not applicable.

## Supplementary Materials

The following supporting information can be downloaded at: https://www.mdpi.com/article/10.3390/molecules28155796/s1, Figure S1: XRD results of MoS2/Cox (x is the addition amount of Co(NO3)2·6H2O, x = 0, 0.1, 0.2, 0.3, 0.5 (mmol)); Figure S2: SEM photograph of MoS2/Cox (x is the addition amount of Co(NO3)2·6H2O, x = 0, 0.1, 0.2, 0.3, 0.5 (mmol)) (a) MoS_2_/Co_0.1_, (b) MoS_2_/Co_0.2_, (c) MoS_2_/Co_0.3_, (d) MoS_2_/Co0.5; Figure S3: CO2 adsorption characteristic curve of MoS2/Cox (x is the addition amount of Co(NO3)2·6H2O, x = 0.1, 0.2, 0.3, 0.5 (mmol)); Figure S4: H2-TPR reduction curve of MoS2/Cox (x is the addition amount of Co(NO3)2·6H2O, x = 0.1, 0.2, 0.3, 0.5 (mmol)); Figure S5: CO2 conversion rate of MoS2, MoS2/Nix and MoS2/Cox catalysts.
