# Supplementary material for: Performance Exploration of Ni-Doped MoS2 in CO2 Hydrogenation to Methanol
Source: Molecules. 2023 Aug 1;28(15):5796. doi: 10.3390/molecules28155796 (PMC10421375; doi:10.3390/molecules28155796)
Supplement: Supplementary file 1 [file molecules-28-05796-s001.zip › molecules-2514961-supplementary.pdf]

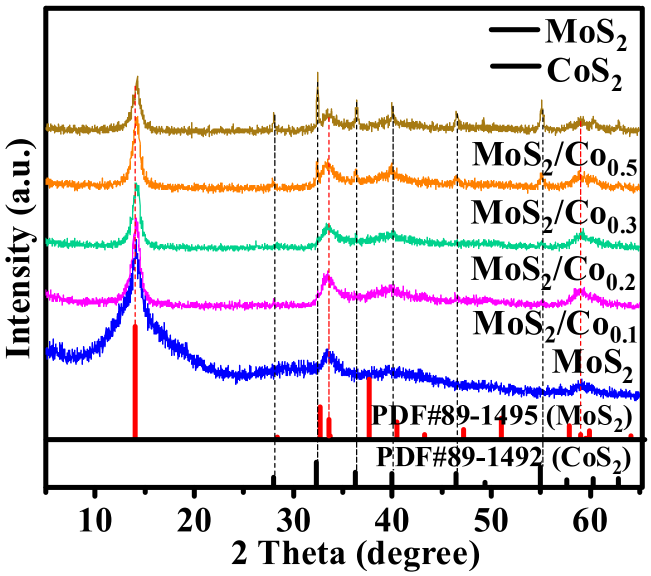

Figure S1. XRD results of MoS_2/Co_x (x is the addition amount of $Co(NO_3)_2·6H_2O$, x=0, 0.1, 0.2, 0.3, 0.5 (mmol)).

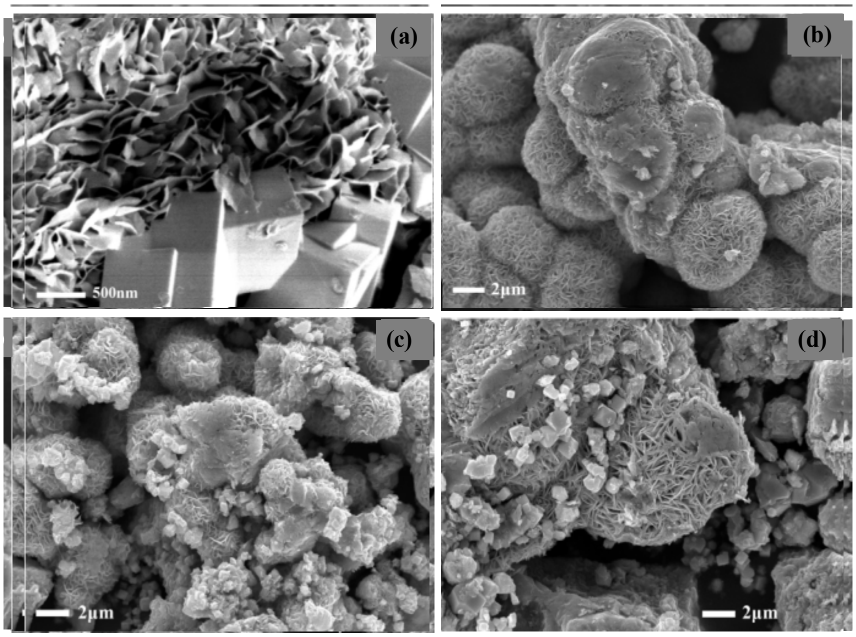

Figure S2. SEM photograph of MoS_2/Co_x (x is the addition amount of $Co(NO_3)_2 \cdot 6H_2O$, x=0, 0.1, 0.2, 0.3, 0.5 (mmol)) **(a)** $MoS_2/Co_{0.1}$, **(b)** $MoS_2/Co_{0.2}$, **(c)** $MoS_2/Co_{0.3}$, **(d)** $MoS_2/Co_{0.5}$.

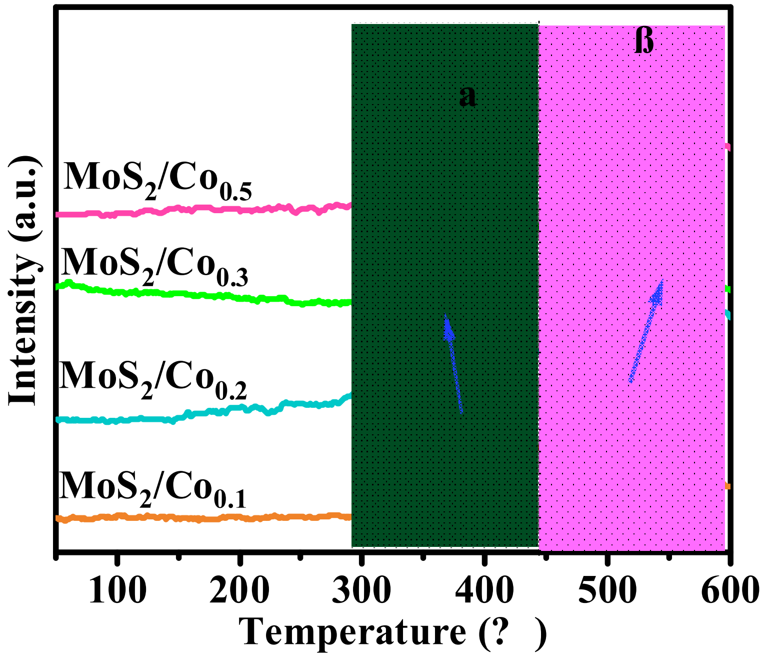

Figure S3. CO_2 adsorption characteristic curve of MoS_2/Co_x (x is the addition amount of $Co(NO_3)_2{\cdot}6H_2O$, x= 0.1, 0.2, 0.3, 0.5 (mmol)).

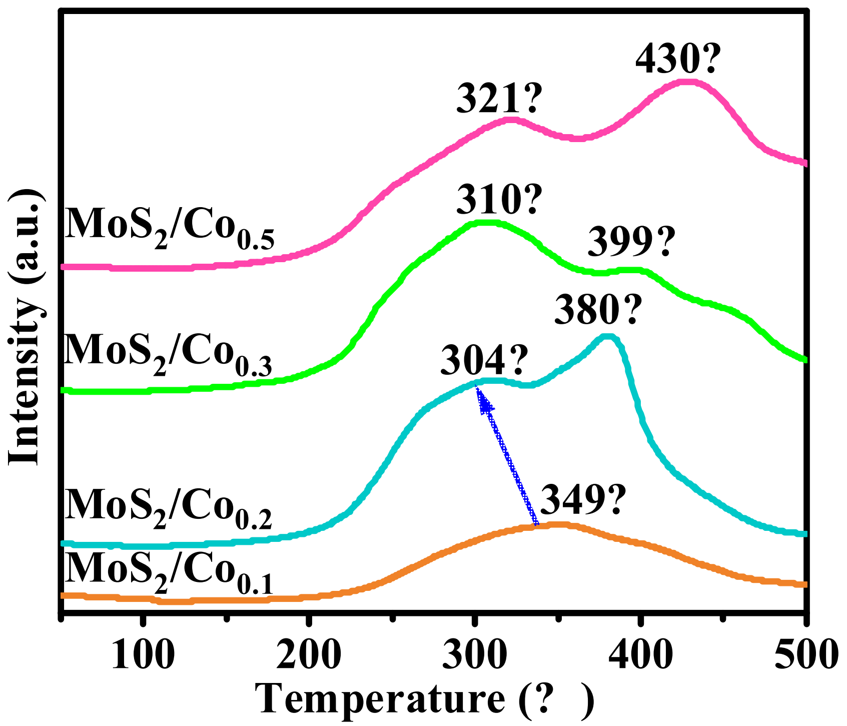

Figure S4. H_2-TPR reduction curve of MoS_2/Co_x (x is the addition amount of $Co(NO_3)_2 \cdot 6H_2O$, x= 0.1, 0.2, 0.3, 0.5 (mmol)).

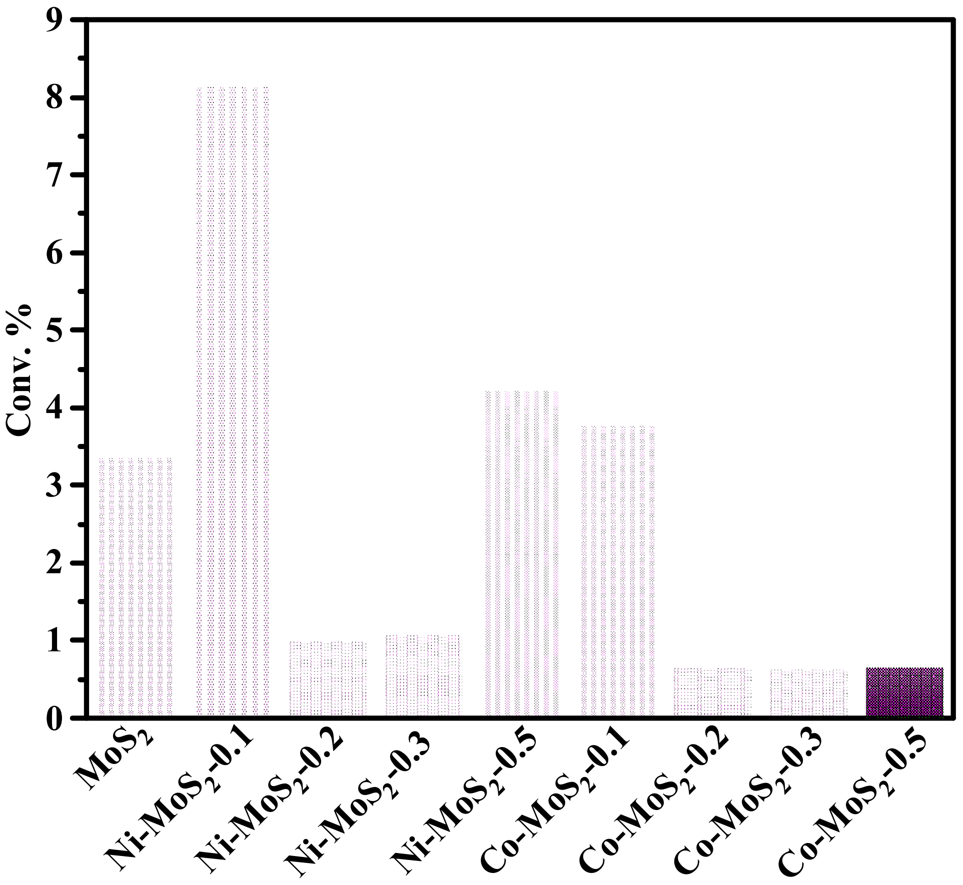

Figure S5. CO_2 conversion rate of MoS_2, MoS_2/Ni_x and MoS_2/Co_x catalysts.
